# Indoxyl Sulfate Inhibits Osteogenesis in Bone Marrow Mesenchymal Stem Cells through the AhR/Hes1 Pathway

**DOI:** 10.3390/ijms25168770

**Published:** 2024-08-12

**Authors:** Chin-Wen Hsieh, Ling-Hua Chang, Yan-Hsiung Wang, Wei-Ting Li, Je-Ken Chang, Chung-Hwan Chen, Mei-Ling Ho

**Affiliations:** 1 Division of Nephrology, Department of Internal Medicine, Pingtung Christian Hospital, Pingtung 900, Taiwan; martinojaz@gmail.com; 2 Graduate Institute of Medicine, College of Medicine, Kaohsiung Medical University, Kaohsiung 807, Taiwan; 3 Orthopaedic Research Center, College of Medicine, Kaohsiung Medical University, Kaohsiung 807, Taiwan; linghua_chang@yahoo.com.tw (L.-H.C.); yhwang@cc.kmu.edu.tw (Y.-H.W.); ruby092294@gmail.com (W.-T.L.); jkchang@cc.kmu.edu.tw (J.-K.C.); 4 Regenerative Medicine and Cell Therapy Research Center, Kaohsiung Medical University, Kaohsiung 807, Taiwan; 5 School of Dentistry, College of Dental Medicine, Kaohsiung Medical University, Kaohsiung 807, Taiwan; 6 Department of Orthopaedics, College of Medicine, Kaohsiung Medical University, Kaohsiung 807, Taiwan; 7 Department of Orthopedics, Kaohsiung Municipal Ta-Tung Hospital, Kaohsiung Medical University, Kaohsiung 801, Taiwan; 8 Institute of Medical Science and Technology, National Sun Yat-sen University, Kaohsiung 804, Taiwan; 9 School of Medicine, College of Medicine, Kaohsiung Medical University, Kaohsiung 807, Taiwan; 10 Ph.D. Program in Biomedical Engineering, College of Medicine, Kaohsiung Medical University, Kaohsiung 807, Taiwan; 11 Graduate Institute of Materials Engineering, College of Engineering, National Pingtung University of Science and Technology, Pingtung 912, Taiwan; 12 Department of Physiology, College of Medicine, Kaohsiung Medical University, Kaohsiung 807, Taiwan; 13 Department of Medical Research, Kaohsiung Medical University Hospital, Kaohsiung 807, Taiwan; 14 Department of Marine Biotechnology and Resources, National Sun Yat-Sen University, Kaohsiung 804, Taiwan; 15 College of Professional Studies, National Pingtung University of Science and Technology, Pingtung 912, Taiwan

**Keywords:** aryl hydrocarbon receptor, indoxyl sulfate, osteogenesis, chronic kidney disease, Hes1

## Abstract

Uremic toxins cause bone disorders in patients with chronic kidney disease (CKD). These disorders are characterized by low turnover osteodystrophy and impaired bone formation in the early stages of CKD. Evidence indicates that the aryl hydrocarbon receptor (AhR) mediates signals that suppress early osteogenic differentiation in bone marrow mesenchymal stem cells (BMSCs). However, whether the AhR mediates the effects of indoxyl sulfate (IS), a uremic toxin, on BMSC osteogenesis remains unclear. We investigated whether IS affects osteogenesis through the AhR/Hes1 pathway. Expression levels of osteogenesis genes (*Runx2*, *Bmp2*, *Alp*, and *Oc*), AhR, and Hes1 were measured in mouse BMSCs (D1 cells). At concentrations of 2–50 μM, IS significantly reduced mineralization, particularly in the early stages of BMSC osteogenesis. Furthermore, IS significantly downregulated the expression of *Runx2*, *Bmp2*, *Oc*, and *Alp*. Notably, this downregulation could be prevented using an AhR antagonist and through *Ahr* knockdown. Mechanistically, IS induced the expression of *Hes1* through AhR signaling, thereby suppressing the transcription of *Runx2* and *Bmp2*. Our findings suggest that IS inhibits early osteogenesis of BMSCs through the AhR/Hes1 pathway, thus suppressing the transcription of *Runx2* and *Bmp2*. Our findings may guide new therapeutic strategies against CKD-related bone disorders.

## 1. Introduction

The global burden of chronic kidney disease (CKD) has been increasing [1]. CKD increases the risk of bone fractures [2] and CKD-related factors such as uremic toxins may thus be involved in the development of bone defects. CKD-related mineral bone disorders manifest as both alterations in bone quantity and quality and abnormalities in bone remodeling [3]. The early stages of CKD involve low-turnover osteodystrophy, which is characterized by defective bone formation [4]; however, the pathophysiology of low-turnover osteodystrophy remains unclear. Understanding its pathophysiology may help optimize therapeutic strategies for early CKD-related mineral bone disorders.

A study involving patients undergoing hemodialysis reported that the levels of the uremic toxin indoxyl sulfate (IS) were elevated and were inversely correlated with those of bone formation markers such as alkaline phosphatase (ALP) and bone-specific ALP [5]. This suggests that IS is associated with bone formation defects in patients with CKD. In vivo evidence supports this association [6], while in vitro studies have indicated that IS reduces the viability and differentiation of bone marrow mesenchymal stem cells (BMSCs) and osteoblasts [7,8]. However, the concentrations of IS used in these studies (mostly ≥100 μM) exceeded the average concentration in patients with CKD (~20 μM in free form [9]). Investigating the osteogenic effects of IS within the concentration range equivalent to circulating concentrations in patients with CKD is necessary to understand the etiopathology of bone defects in CKD.

The aryl hydrocarbon receptor (AhR) is a ligand-activated transcription factor that is involved in various biological processes, such as liver development, reproduction, and immune function [10,11,12]. The AhR regulates the expression of osteogenic genes during the early stages of differentiation in multipotent BMSCs [13]; this protein also mediates the toxin-induced impairment of osteogenesis [14]. At nanomolar concentrations, IS was reported to be a potent AhR ligand [15]. A study indicated that IS at a concentration of 500 μM could induce osteogenic defects in osteoblasts through nongenomic AhR signaling [16]. However, whether IS at concentrations similar to those in patients with CKD can cause osteogenic defects through the AhR remains unclear. Furthermore, the effects of IS on AhR-targeted genes and early osteogenic differentiation in BMSCs have yet to be clarified.

The basic helix–loop–helix transcription repressor hairy/enhancer of split-1 (Hes1) plays pivotal roles in embryogenesis [17], including the inhibition of neuronal cell differentiation [18] and the suppression of chondrogenesis [19]. However, the effects of Hes1 on osteogenesis remain debatable. A study indicated that Hes1 inhibits osteoblastogenesis in stromal cells through protein–protein interaction between Hes1 and runt-related transcription factor 2 (Runx2) [20]. Another study implicated the Notch/Hes1 pathway in the promotion of bone morphogenetic protein 2 (Bmp2)-induced osteoblastic differentiation [21]. The mechanisms underlying the associations among Hes1, Runx2, and Bmp2 in osteogenic cells remain unclear. The AhR has been reported to induce Hes1 expression in mammary carcinoma and liver cells [22,23]. However, whether the AhR induces Hes1 expression in BMSCs or affects BMSC osteogenesis through Hes1 expression remains to be clarified. Accordingly, the objective of this study was to understand the mechanism through which the AhR regulates osteogenic gene expression; we analyzed the expression of Notch receptors (*Notch1–4*) and canonical Notch-targeted genes—*Hes1* and hairy/enhancer of split with YRPW motif 1 (*Hey1*)—in BMSCs. Results demonstrated that IS inhibits the early osteogenesis of BMSCs through the AhR/Hes1 pathway, thus suppressing the transcription of *Runx2* and *Bmp2*. Our findings may clarify the molecular mechanisms underlying IS/AhR-mediated osteogenic defects.

## 2. Results

### 2.1. IS Suppresses the Mineralization of D1 Cells

**Figure 1:**
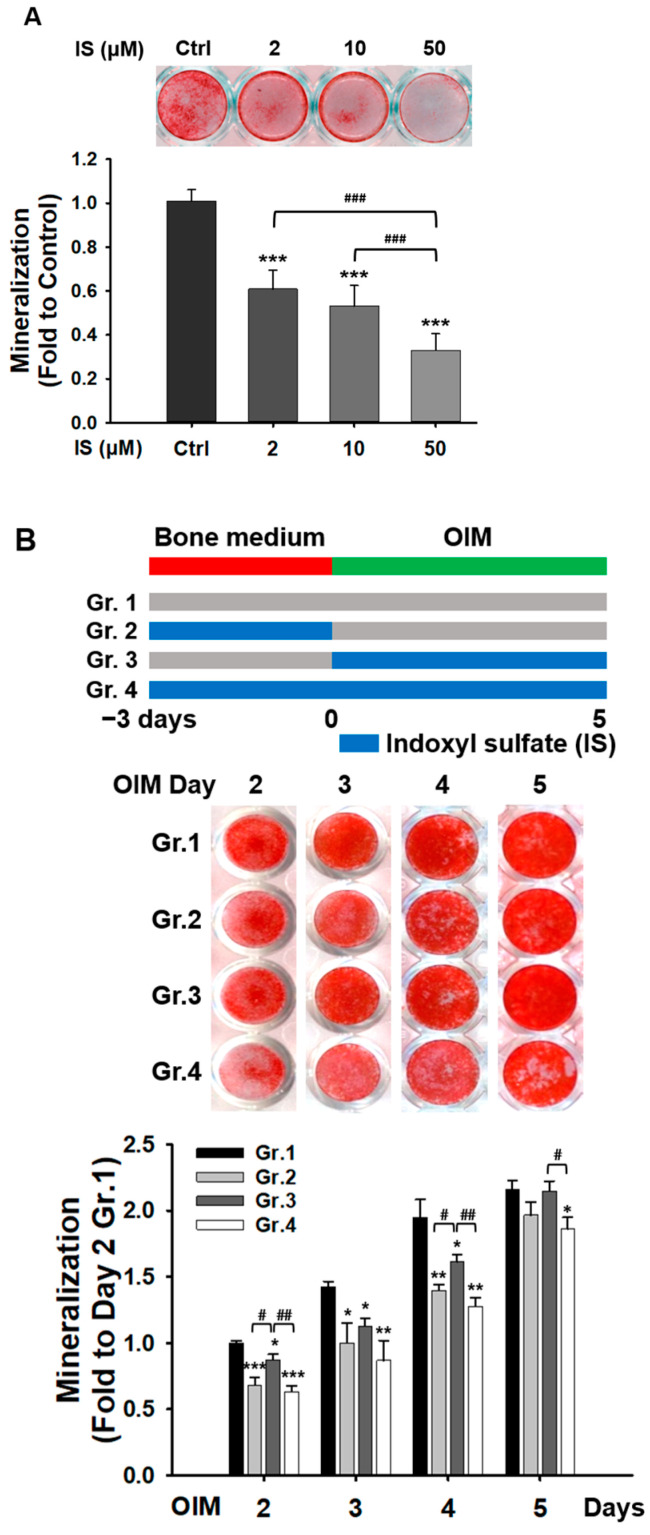
Indoxyl sulfate (IS) reduced the mineralization of D1 cells. D1 cells cultured in osteoinduction medium for (**A**) 3 and (**B**) 2–5 days were stained with Alizarin Red S (ARS). (**A**) IS (2–50 mM) reduced the level of D1 cell mineralization in a dose-dependent manner. (**B**) Effects of IS (50 μM) on various stages of osteogenesis in D1 cells. The upper panel depicts the study design and the lower panel presents quantitative data pertaining to D1 cell mineralization. Data are presented as mean ± standard deviation values (* *p* < 0.05, ** *p* < 0.01, *** *p* < 0.001; * compared with the control. # *p* < 0.05, ## *p* < 0.01, ### *p* < 0.001; # compared with 50 mM IS (**A**) and group 3 (**B**); analysis of variance). OIM, osteoinduction medium.

To determine the concentrations of IS that did not affect the viability of D1 cells, we conducted lactate dehydrogenase cytotoxicity and 3-[4,5-dimethylthiazol-2-yl]-2,5-diphenyl-tetrazolium bromide assays. The results indicated that IS at concentrations of ≤50 μM exerted no effects on cell viability during 1–3 days of culture (Figures S1 and S2). Consequently, we evaluated the osteoblastic potential of D1 cells at IS concentrations of 2–50 μM. Alizarin Red S (ARS) staining and quantitative analysis performed to assess osteoblastic potential revealed that IS dose-dependently suppressed mineralization in the D1 cells (Figure 1A). To determine the primary stage at which IS affects mineralization, we treated the D1 cells with 50 μM IS during early, late, or both stages of differentiation (Figure 1B, upper panel). The results revealed that IS significantly reduced the levels of mineralization during the early stages of differentiation (Figure 1B, lower panel). This thus indicates that IS may inhibit the in vitro osteoblastic activity of D1 cells primarily during the early stages of differentiation. Accordingly, subsequent experiments were performed, focusing on the effects of 50 μM IS on D1 cells during the early stages of differentiation.

### 2.2. IS Downregulates the Expression of Osteogenic Markers in D1 Cells 

**Figure 2:**
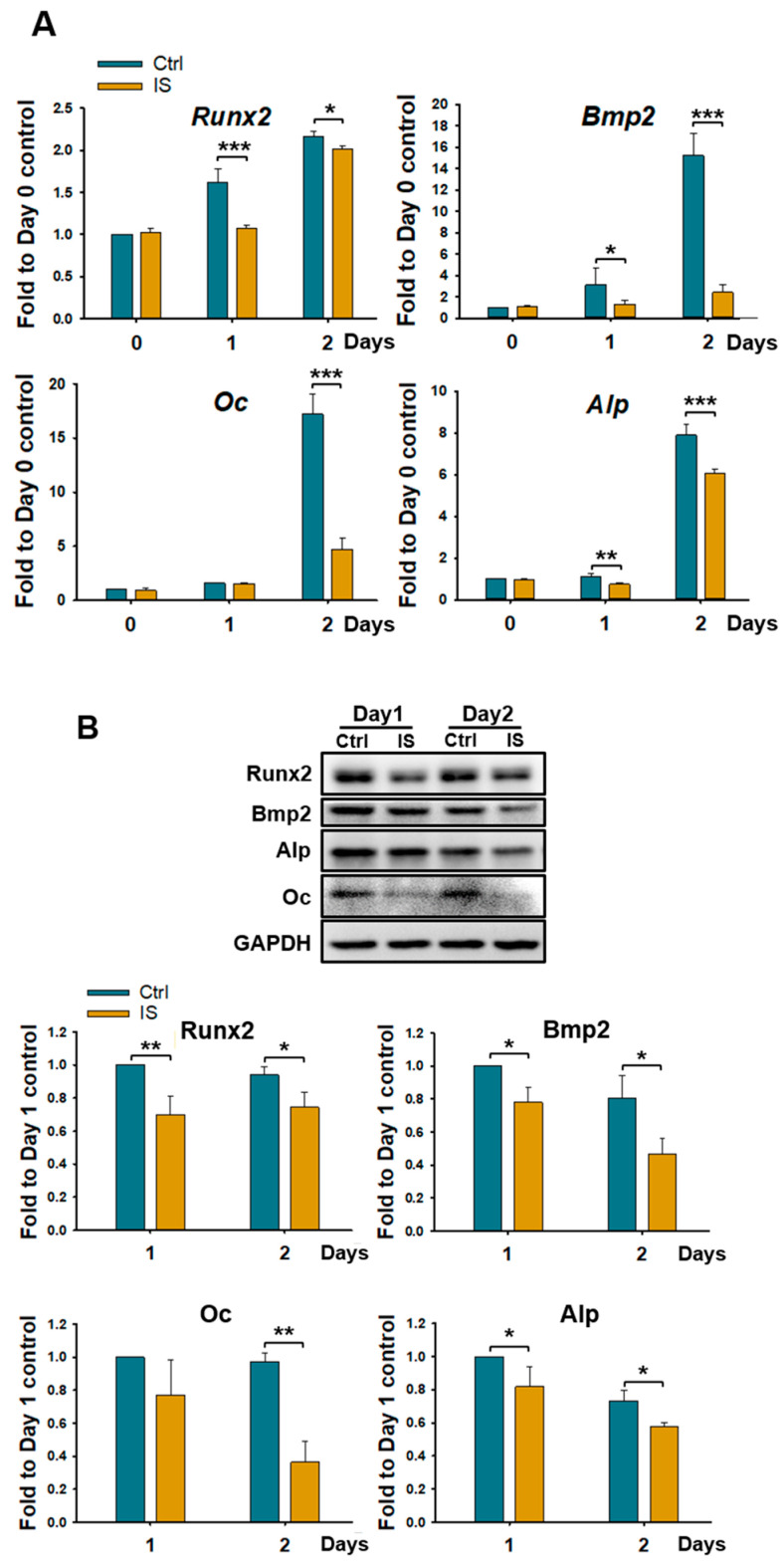
IS downregulated the expression of osteogenic markers in D1 cells. (**A**) mRNA levels of runt-related transcription factor 2 (*Runx2*), bone morphogenetic protein 2 (*Bmp2*), osteocalcin (*Oc*), and alkaline phosphatase (*Alp*) in D1 cells treated with IS (50 μM) for 1 and 2 days or left untreated. (**B**) Protein levels of osteogenic markers. Glyceraldehyde 3-phosphate dehydrogenase (*GAPDH*) was used as a housekeeping gene control. Images depict the results of Western blotting (upper and lower panels). Three independent experiments, each in triplicate, were performed. Data are presented as mean ± standard deviation values (* *p* < 0.05, ** *p* < 0.01, and *** *p* < 0.001; * compared with the control; Student *t*-test).

We investigated whether IS affects the expression of the osteogenic markers *Runx2*, *Bmp2*, *Alp*, and *Osteocalcin* (*Oc*). Quantitative real-time polymerase chain reaction (qRT-PCR) revealed significant reductions in the mRNA levels of these markers in the IS-treated cells on days 1 and 2 compared with the levels in the control cells (Figure 2A). Western blotting further confirmed that, unlike the vehicle, IS reduced the levels of these markers in the D1 cells (Figure 2B). Therefore, IS downregulates the expression of osteogenic genes during the early stages of differentiation in D1 cells.

### 2.3. IS Activates AhR Signaling

**Figure 3:**
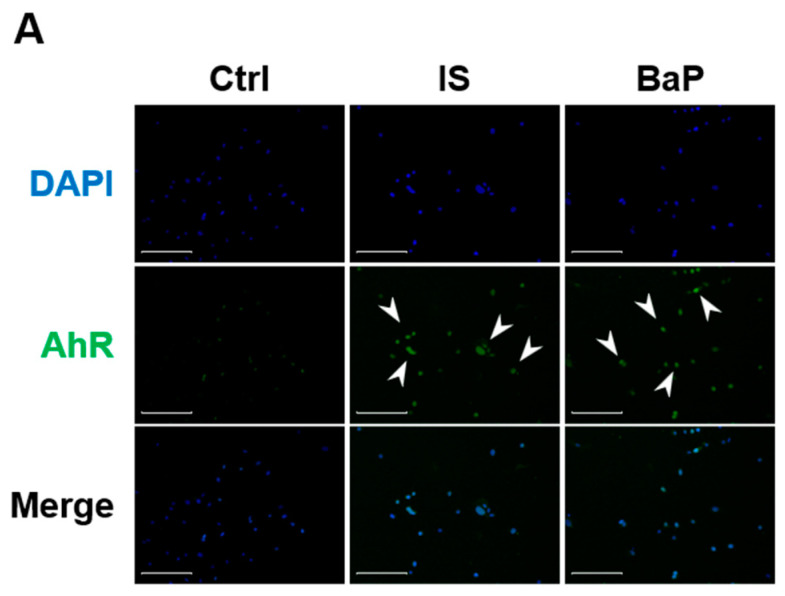

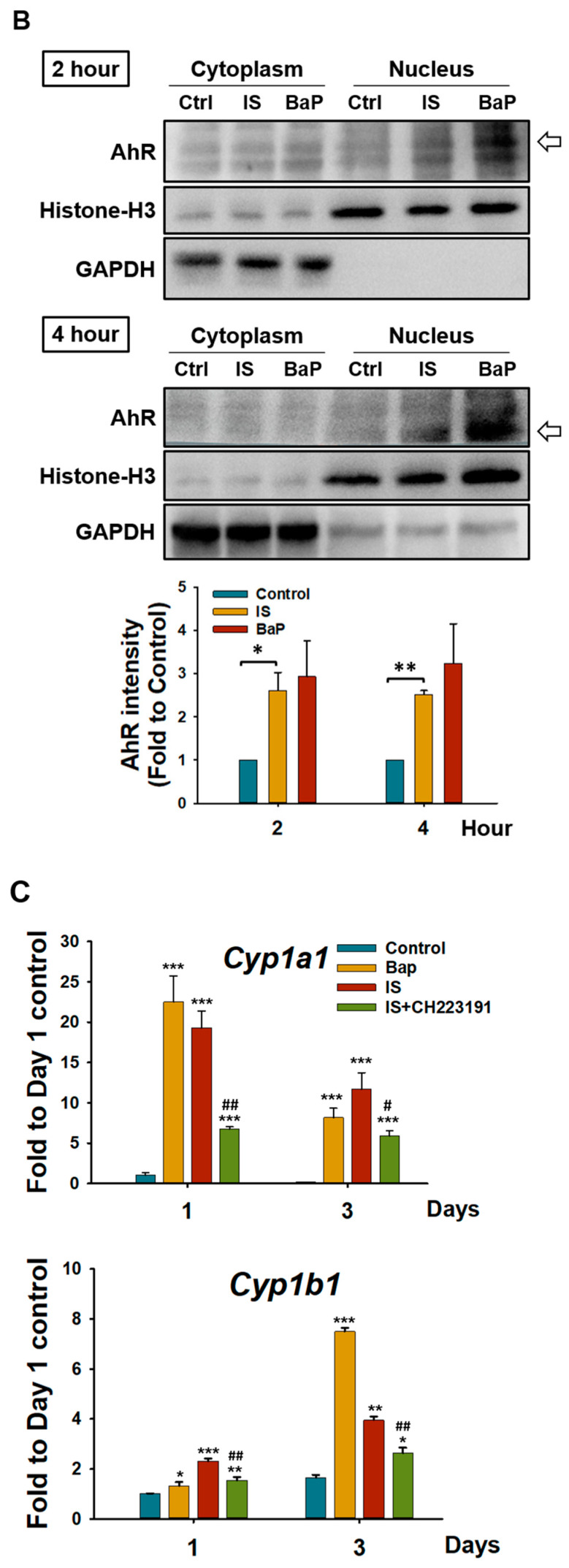

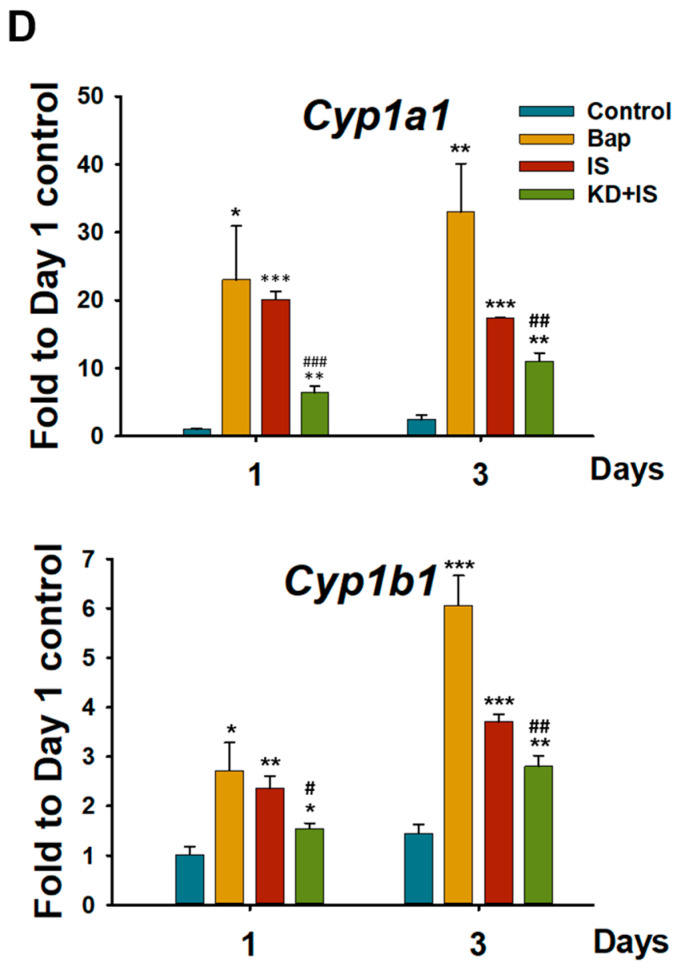
IS activated the aryl hydrocarbon receptor (AhR) signaling pathway. (**A**) Immunofluorescence staining of AhR in IS-treated (for 1 h) D1 cells. Scale bar = 150 µm; magnification = 20×. The nuclei were stained blue through DAPI staining. (**B**) Effect of IS on the translocation of AhR from the cytoplasm to the nucleus in D1 cells. Cells were treated with a vehicle control, IS, or benzo[a]pyrene (BaP; an AhR agonist serving as the positive control) for 2 and 4 h. Total nuclear and cytosolic fractions were isolated and AhR levels were measured through Western blotting. GAPDH and histone-H3 were used as marker proteins for nonnuclear and nuclear fractions, respectively. The lower panel depicts the quantitation of nuclear AhR from Western blotting. (**C**,**D**) mRNA levels (measured through quantitative real-time polymerase chain reaction [qRT-PCR]) for cytochrome P450 family 1 subfamily A member 1 (*CYP1A1*) and cytochrome P450 family 1 subfamily B member 1 (*CYP1B1*) in D1 cells were compared among control, BaP-treated, and IS-treated cells subjected or not subjected to AhR antagonist (CH-223191; 10 μM) treatment (**C**) or *Ahr* silencing (KD) (**D**). Data are presented as mean ± standard deviation values (* *p* < 0.05, ** *p* < 0.01, *** *p* < 0.001; * compared with the control. # *p* < 0.05, ## *p* < 0.01, ### *p* < 0.001; # compared with the IS-treated group; analysis of variance).

To elucidate the role of AhR in mediating the effects of IS on D1 cell mineralization, we first investigated whether IS activates AhR signaling. The results indicated that IS induced the nuclear localization of AhR (Figure 3A). Western blotting revealed that IS increased the nuclear abundance of AhR compared with its level in the control cells (Figure 3B). Moreover, IS upregulated the expression of the primary AhR target gene cytochrome P450 family 1 subfamily A member 1 (*Cyp1A1*) and cytochrome P450 family 1 subfamily B member 1 (*Cyp1B1*). However, their expression could be inhibited by the AhR antagonist CH-223191 (Figure 3C) and through *Ahr* knockdown (Figure 3D). To downregulate the expression of *AhR*, the D1 cells were transfected with AhR-small interfering RNA (siRNA) (Figure S3). These results indicate that IS induces AhR downstream signaling in D1 cells. Therefore, IS-induced AhR activation may mediate the regulation of osteogenic genes and the mineralization of D1 cells.

### 2.4. IS Impairs Osteogenesis by Inducing AhR

**Figure 4:**
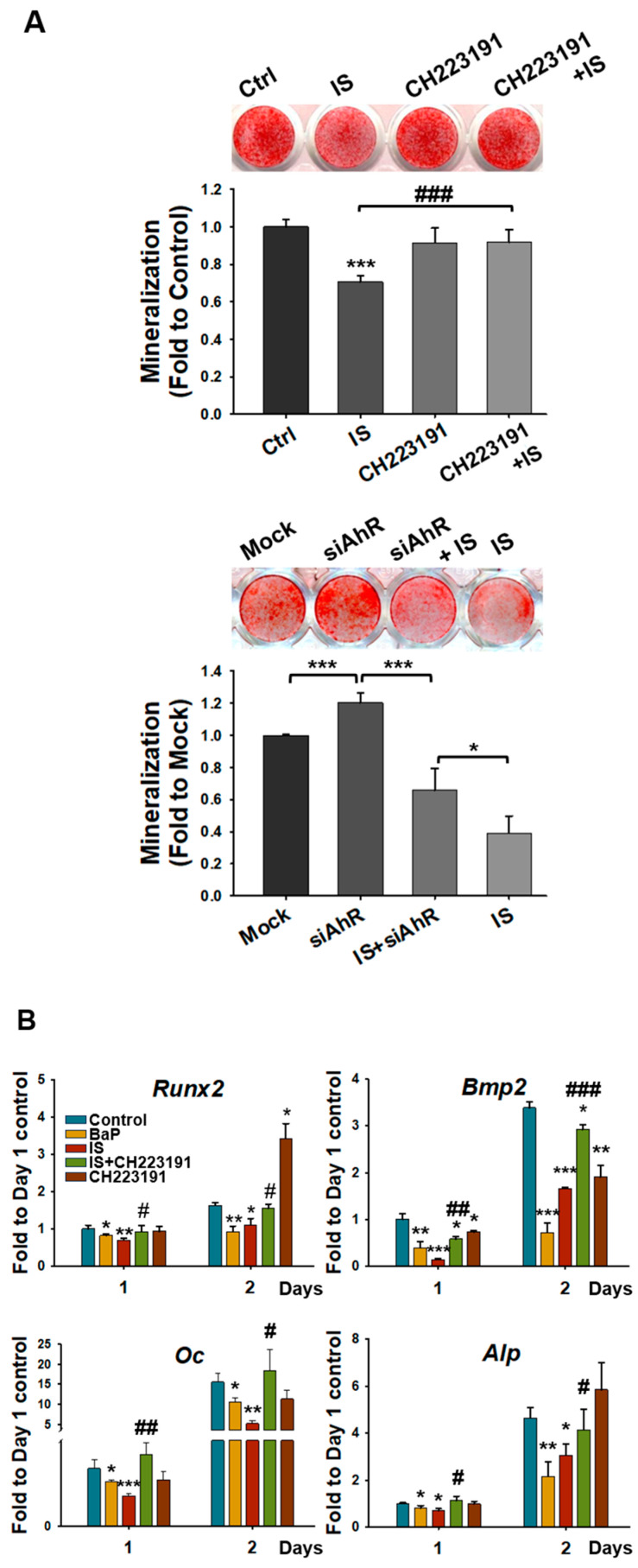

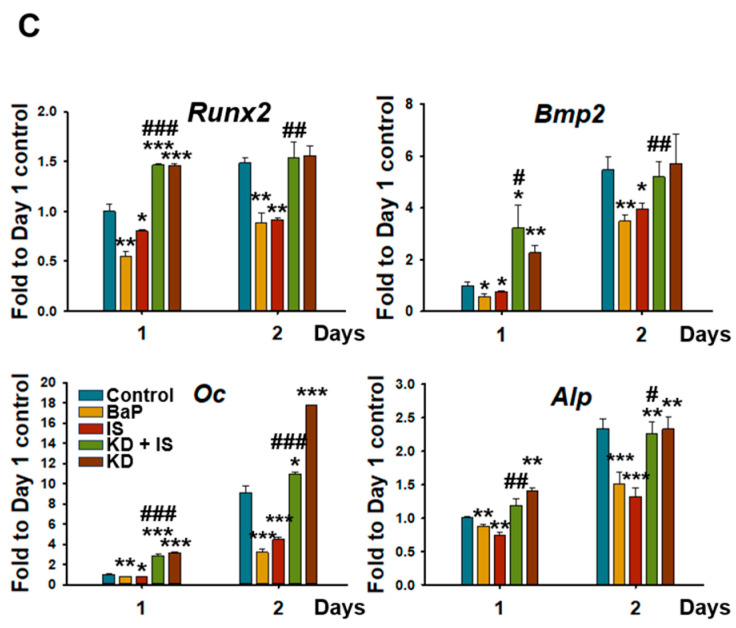
IS suppressed the mineralization of D1 cells through AhR. (**A**) AhR antagonist (CH-223191; 10 μM; **upper panel**) and *Ahr* knockdown (**lower panel**) completely or partially, respectively, prevented the IS-mediated suppression of D1 cell mineralization. D1 cells were transfected with the vector of control-siRNA or AhR-siRNA for 48 h and cultivated in a bone medium for another 3 days. Mineralization was assessed through the ARS staining of D1 cells cultured in osteoinduction medium for 3 days. (**B**,**C**) mRNA levels of osteogenic markers in D1 cells were determined through qRT-PCR. D1 cells were treated with IS (50 μM) with or without CH-223191 (10 μM) (**B**) or with or without AhR-siRNA (KD) (**C**) for 1 and 2 days. Data are presented as mean ± standard deviation values (* *p* < 0.05, ** *p* < 0.01, *** *p* < 0.001; * compared with the control. # *p* < 0.05, ## *p* < 0.01, ### *p* < 0.001; # compared with the IS-treated group; analysis of variance).

To clarify how the AhR pathway mediates the IS-induced reduction in osteogenesis, we performed ARS staining. The inhibition of AhR activity with an antagonist and through *Ahr* knockdown mitigated the IS-mediated reduction in the level of extracellular matrix calcification in the D1 cells (Figure 4A). Furthermore, qRT-PCR revealed that the AhR antagonist (Figure 4B) and *Ahr* knockdown (Figure 4C) prevented the IS-mediated downregulation of the expression of the osteogenic markers. These results thus demonstrate that IS suppresses osteogenic differentiation and downregulates osteogenic gene expression through the AhR pathway.

### 2.5. IS Upregulates the Expression of Hes1 through AhR

**Figure 5:**
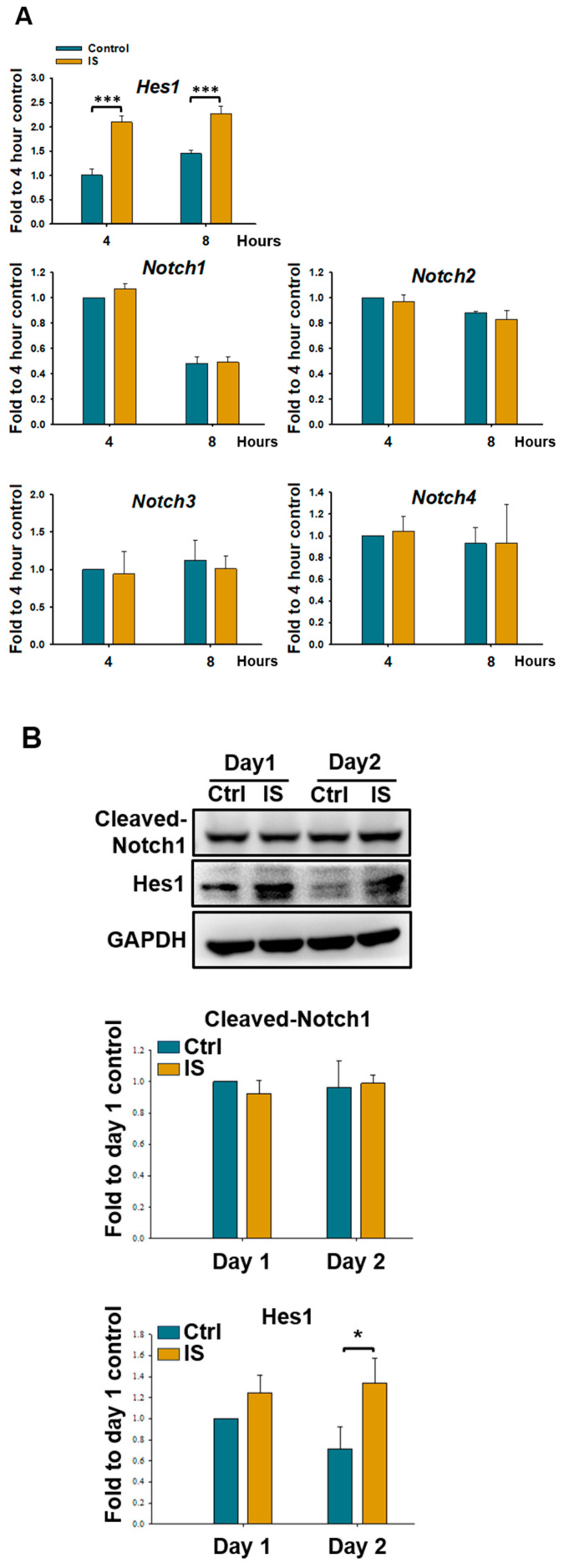

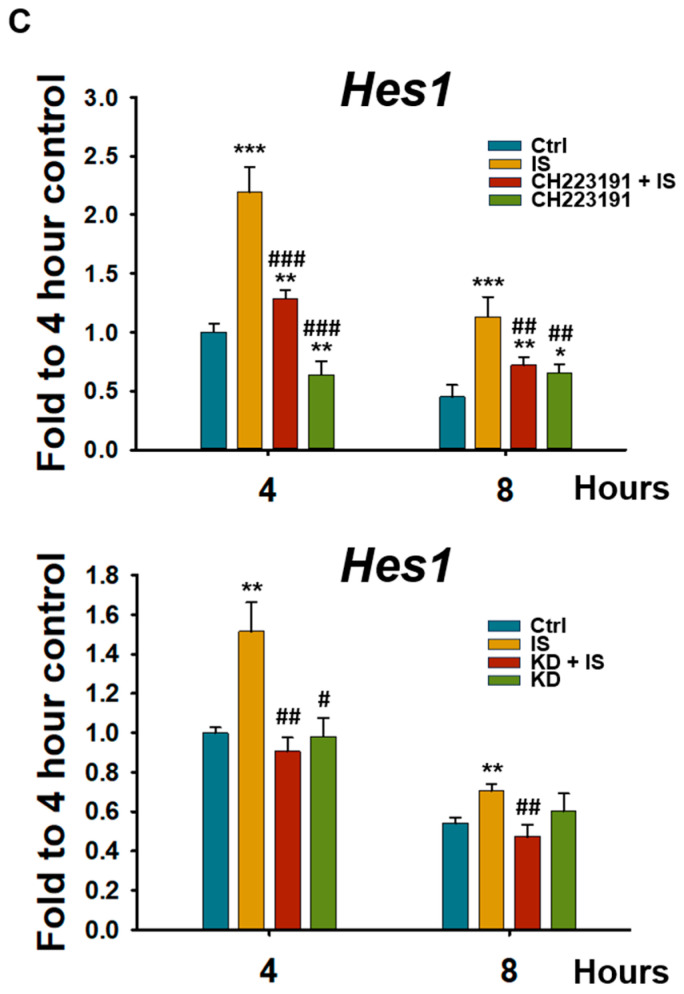
IS upregulated the expression of hairy/enhancer of split-1 (*Hes1*) through AhR. (**A**) qRT-PCR revealed the mRNA levels of Notch receptors and *Hes1* in D1 cells treated with IS for at least 4 and 8 h or left untreated. (**B**) Western blotting revealed the protein levels of cleaved-Notch1 and Hes1 in D1 cells treated with IS or a vehicle control for 1 and 2 days. (**C**) qRT-PCR indicated the level of *Hes1* expression in control or IS-treated D1 cells subjected to AhR antagonist (CH-223191) treatment (upper panel) or *Ahr* knockdown (KD) (lower panel) for 4 and 8 h. Data are presented as mean ± standard deviation values (*n* = 3; * *p* < 0.05, ** *p* < 0.01, *** *p* < 0.001; * compared with the control. # *p* < 0.05, ## *p* < 0.01, ### *p* < 0.001; # compared with the IS-treated group; Student *t*-test (**A**,**B**) and analysis of variance (**C**)).

To determine whether IS regulates the expression of *Hes1* and elucidate the underlying mechanism, we evaluated the expression levels of Hes1, Notch receptors (Notch1–4), and another Notch target gene (*Hey1*) during the early stages of differentiation in D1 cells. The results indicated that IS did not affect the mRNA level of *Notch1–4* (Figure 5A) or *Hey1* (Figure S4) during the initial 4–8 h of differentiation. However, IS significantly increased the mRNA level of *Hes1* during this period (Figure 5A). Western blotting revealed an increase in the level of Hes1 but no changes in that of cleaved-Notch1 (a downstream signal mediator of the Notch pathway) after IS treatment (Figure 5B). These results indicate that IS upregulates the expression of *Hes1* without affecting the Notch signaling pathway. Furthermore, AhR antagonist treatment and *Ahr* knockdown prevented IS-induced changes in *Hes1* expression (Figure 5C). Therefore, IS-induced AhR activation appears to upregulate the expression of *Hes1* without affecting the Notch pathway. This thus signifies that Hes1 may be involved in the IS/AhR-mediated suppression of osteogenesis in D1 cells during the early stages of differentiation.

### 2.6. Hes1 Downregulates the Expression of Runx2 and Bmp2

**Figure 6:**
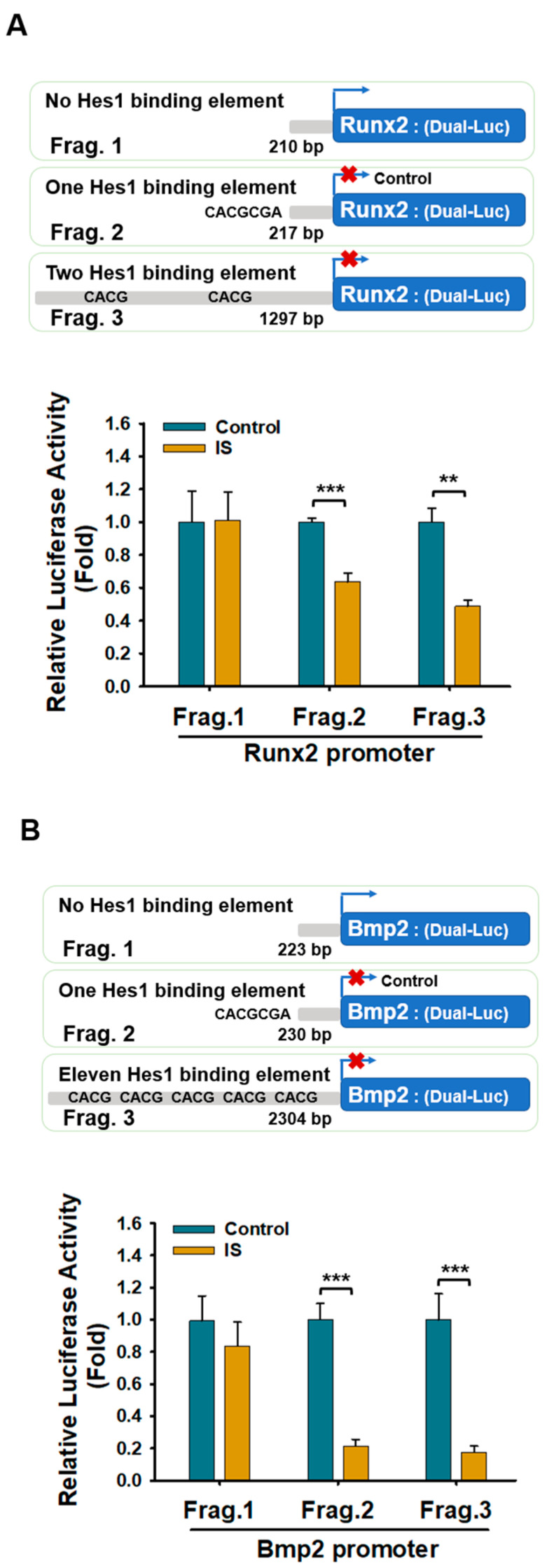
Hes1 suppressed the activity of the *Runx2* and *Bmp2* promoters. (Upper panel: (**A**,**B**)) *Runx2* (**A**) and *Bmp2* (**B**) promoters featuring various Hes1-binding sites. (Lower panel: (**A**,**B**)) D1 cells were transfected with vectors of the specified promoter constructs for 24 h. Subsequently, the cells were treated either with IS or a vehicle control for another 24 h. The cells were then subjected to a dual-luciferase glow assay to measure the activity of luciferase. The results represent data from three independent experiments with consistent outcomes. Data are presented as mean ± standard deviation values (** *p* < 0.01, and *** *p* < 0.001; * compared with the control; Student *t*-test).

To test the hypothesis that Hes1 would suppress osteogenesis by downregulating the expression of *Runx2* and *Bmp2*, we first identified potential Hes1-binding N-boxes in the promoter regions of *Runx2* and *Bmp2* by using PROMO (version 3.0). The findings supported our hypothesis. Furthermore, to investigate whether Hes1 regulates transcription by binding to these Hes1-response elements in the promoters of *Runx2* and *Bmp2*, we conducted a dual-luciferase reporter assay. The results revealed that IS significantly suppressed the activity of the *Runx2* and *Bmp2* promoters in D1 cells transfected with plasmids containing 2 and 11 Hes1-binding elements, respectively, compared with their activity in cells transfected with plasmids lacking these elements (Figure 6A,B). These findings indicate that Hes1 suppresses the activity of the *Runx2* and *Bmp2* promoters by binding to corresponding N-boxes in the promoter regions.

### 2.7. Hes1 Directly Binds to the Promoters of Runx2 and Bmp2

**Figure 7:**
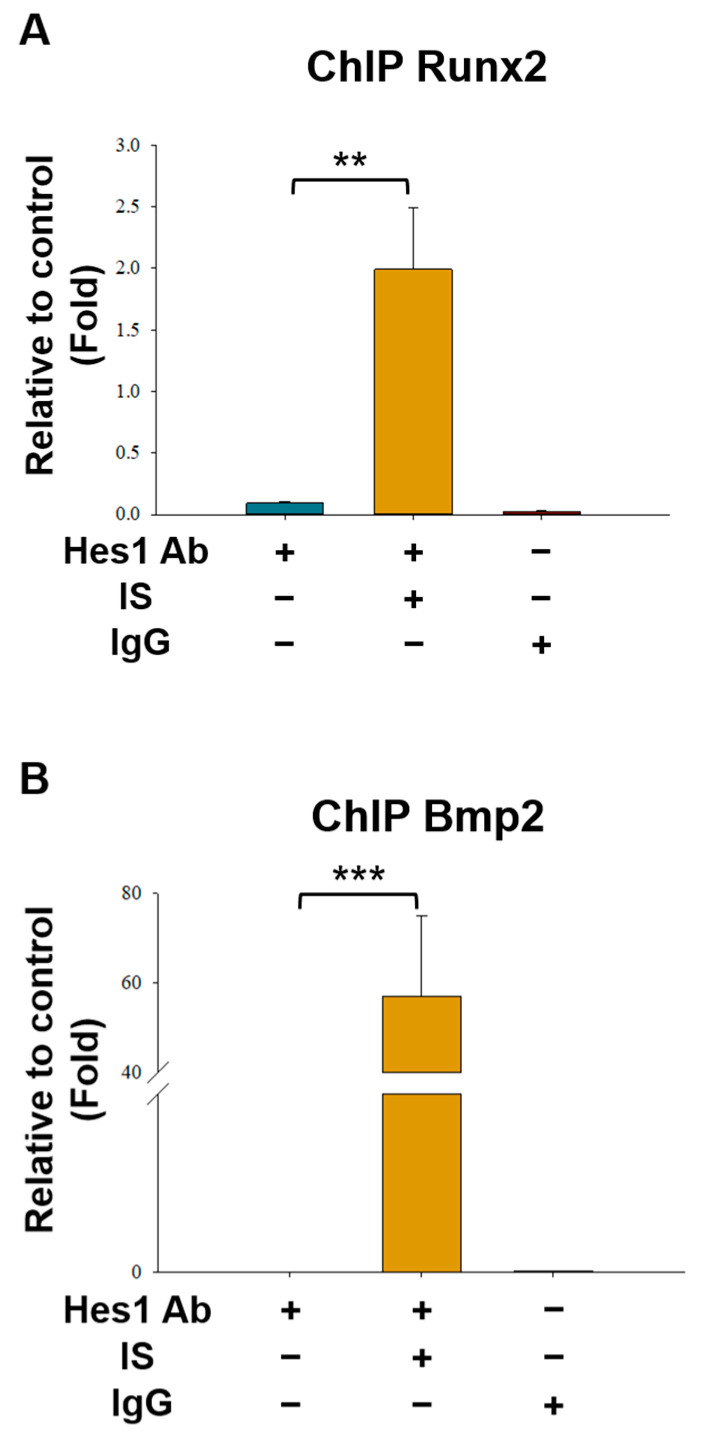
Hes1 is directly bound to the promoters of *Runx2* and *Bmp2*. A chromatin immunoprecipitation (ChIP) assay was performed using chromatin isolated from D1 cells; anti-Hes1 antibodies were used for immunoprecipitation. After 24-h IS treatment (antibodies used in ChIP: those against IS + Hes1) or no treatment (antibodies used in ChIP: those against Hes1 only), DNA from D1 cells was analyzed through quantitative PCR performed using primers targeting the Hes1-binding sites in the promoters of (**A**) *Runx2* and (**B**) *Bmp2*. Immunoglobulin (Ig) G served as the negative control, whereas the input (total DNA extract) served as the positive control. The experiments were performed in triplicate. Data are presented as mean ± standard deviation values (** *p* < 0.01, and *** *p* < 0.001; analysis of variance).

To determine whether Hes1 directly binds to the *Runx2* and *Bmp2* promoters, we performed a chromatin immunoprecipitation (ChIP) assay (by using Hes1 antibodies for the immunoprecipitation of D1 cell lysate), followed by real-time PCR. The results revealed that Hes1 binds to the promoters of *Runx2* and *Bmp2* in the D1 cells. This binding was confirmed through ChIP and real-time PCR (performed using specific primers for the *Runx2* and *Bmp2* promoters; Figure 7A,B). The results of the reporter and ChIP assays confirmed that Hes1 suppresses the activity of the *Runx2* and *Bmp2* promoters by directly binding to corresponding N-boxes at the transcription start site.

## 3. Discussion

This study found that IS, at clinically relevant concentrations, suppressed osteogenesis during the early stages of differentiation in BMSCs. According to our review of the literature, our study is the first to demonstrate that IS upregulates the expression of *Hes1* through genomic AhR signaling, inhibiting the expression of *Bmp2* and *Runx2* without interfering with the Notch signaling pathway. Furthermore, our study is the first to examine the involvement of the AhR–Hes1–BMP2 regulatory axis in BMSC osteogenesis.

AhR signaling operates through either the genomic or nongenomic pathway. In the genomic pathway, the ligand–AhR complex translocates into the nucleus and regulates the transcription of specific genes. In addition to genomic AhR signaling, nongenomic signaling activates protein kinases and phosphatases [24], thereby regulating the functions of various proteins and mediating the biological or harmful effects of the AhR. Notably, the AhR has been implicated in developmental and degenerative bone disorders [25,26]. The AhR inhibits osteogenesis through its effects on beta-catenin and estrogen receptor alpha [27,28]. Moreover, IS can induce the AhR to downregulate Runx2 expression and osteogenesis by suppressing the extracellular signal-regulated kinase and p38 mitogen-activated protein kinase (MAPK) pathways [16]. Thus, the AhR appears to prevent osteogenesis through the nongenomic pathway. However, the role of the AhR’s genomic pathway in the development of IS-induced osteogenic defects remains unclear. A study on smoke toxin reported that AhR mediates smoke-induced toxic effects, downregulates *Bmp2* expression, and suppresses osteogenesis by elevating the mRNA level of *Hey1* [29]; these findings imply that AhR signaling operates through the genomic pathway in the given context. In this study, we observed that IS functions through the AhR to simultaneously upregulate the transcription of *Hes1* but not *Hey1* and downregulate the expression of osteogenic genes. Therefore, the inhibitory effect of IS on BMSC osteogenesis is mediated through the AhR/Hes1 genomic pathway rather than the previously indicated nongenomic pathway [16].

The AhR plays a pivotal role in maintaining the stem cell pool and modulating early osteogenic differentiation [13]. We demonstrated that the AhR mediates the inhibitory effect of IS on osteogenesis during the early stages of BMSC differentiation. This finding suggests that uremic toxins suppress bone formation in patients with CKD by downregulating the expression of early osteogenic genes in BMSCs, including Hes1 and BMP2. Further research on the early effects of uremic toxins on BMSCs is warranted to understand the basic biology and guide clinical treatment of CKD-related osteogenic defects. 

Hes1, the basic helix–loop–helix transcription factor, regulates cellular functions during the early stages of development, including the cell cycle, proliferation, and differentiation [30,31]. In the context of osteogenic differentiation, Hes1 serves as both a transcriptional activator and a repressor [32,33]. A previous study indicated that Notch/Hes1 signaling counteracts Wnt-mediated osteoblastogenesis and ALP activity in ST-2 cells [33]. Moreover, another study suggested that Hes1 inhibits the function and differentiation of osteoblasts by regulating the function of Runx2 [34]. However, a study demonstrated that Hes1 can also activate osteogenic differentiation in prostate cancer bone metastatic cells [32]. Our study revealed that Hes1 inhibits the osteogenesis of BMSCs. Our findings differ from those of the previous study [32], possibly because of the variations in cell lines and culture conditions. Further research is warranted to investigate the transcriptional properties of Hes1 in osteogenesis, focusing on context-dependent mechanisms.

Although Hes1 can inhibit osteoblastogenesis, the direct association between Hes1 and BMP2 signaling remains unclear [33]. BMP2 is an upstream regulator of Hes1 [35,36]. To date, evidence has yet to be provided regarding whether Hes1 regulates the expression of BMP2. We observed that Hes1 functions as an upstream regulator and downregulates the transcription of not only *Bmp2* but also *Runx2* in BMSCs. This finding clarifies another mechanism through which the Hes1/BMP2 pathway regulates osteogenesis in BMSCs. Furthermore, the AhR mediates the induction of Hes1 expression in malignant and liver cells [22,23,37]. However, whether the AhR/Hes1 pathway regulates osteogenic differentiation in BMSCs remains to be explored. Our study appears to be the first to demonstrate that IS downregulates the expression of osteogenic genes—*Bmp2* and *Runx2*—through the AhR/Hes1 pathway. Additionally, our study highlights the significance of this pathway in osteogenesis and its potential role in CKD-related bone disorders. The mechanisms through which the AhR and Hes1 are regulated must be explored further to better understand CKD-related bone disorders.

The AhR upregulates Notch receptors and their primary effector, Hes1, to regulate male fertility [12]. Because Notch signaling is involved in osteoblast differentiation [38,39], AhR might have influenced Notch signaling and Hes1 in our cellular model. However, the expression of *Hes1* was significantly upregulated, whereas that of *Notch1–4* mRNAs remained unchanged or exhibited only a subtle change 24 h after IS treatment, suggesting Hes1 to be the primary target activated by the AhR independently from the Notch signaling pathway. In addition to the effect of IS, direct upregulation of *Hes1* expression through the AhR, independent from Notch, was observed in human mammary carcinoma cells [22]. The mechanisms underlying the regulation of *Hes1* expression through the AhR’s genomic pathway should be investigated in future studies.

Evidence suggests that 200 mM IS inhibits osteogenesis in BMSCs primarily by reducing their viability and proliferation [8,40]. However, IS concentrations above 200 μM far exceed the average concentration in patients with CKD (approximately 20 μM) [9]. In another study, osteoblastic MC3T3-E1 cells treated with IS at concentrations over 500 μM showed inhibition of osteogenesis through the AhR-MAPK nongenomic pathway [16]. These high IS concentrations used in the aforementioned studies might not have accurately reflected the clinical effects of IS on bone. Accordingly, in this study, we used IS concentrations of 2–50 μM, mimicking the circulating concentrations of IS in patients with CKD. Our results reveal that even at these lower clinically relevant concentrations, IS inhibits BMSC osteogenesis. Hence, BMSCs may be the primary target for the IS-induced suppression of osteogenesis, which eventually causes bone defects in patients with CKD. Moreover, our results provide evidence for the role of uremic toxins in developing osteogenic defects in early-stage CKD, where patients often exhibit relatively low levels of uremic toxins.

The limitation of our study is that we solely utilized in vitro cell line models. Future in vivo animal studies are warranted to further validate the involvement of the AhR/Hes1 genomic pathway in the IS-induced bone defects associated with CKD.

In conclusion, our study may be the first to report that IS at clinical concentrations (circulating concentrations in patients with CKD) disrupts the early stages of osteogenic differentiation in mouse BMSCs. Furthermore, Hes1 is a key mediator in the IS-induced AhR pathway, suppressing osteogenesis. According to our review of the literature, this study is the first to reveal the AhR/Hes1/BMP2 pathway in BMSCs. Understanding the mechanisms of osteogenic defects in patients with CKD is crucial for improving the quality of care. Further research on the precise mechanisms underlying the IS-induced suppression of osteogenesis is necessary to guide innovative therapeutic approaches for CKD-related bone turnover diseases.

## 4. Materials and Methods

### 4.1. Cell Culture and Treatment

This study used D1 cells (CRL-12424; American Type Culture Collection Center, Manassas, VA, USA) obtained from a mesenchymal stem cell line that was cloned from the bone marrow cells of BALB/c mice. The D1 cells were cultured in bone medium comprising Dulbecco’s modified Eagle’s medium (Gibco BRL, Thermo Fisher Scientific, Waltham, MA, USA, Cat# 31600034) that was supplemented with 10% charcoal-stripped fetal bovine serum (Sigma-Aldrich, St. Louis, MO, USA, Cat# TMS-013-BKR), 0.5% penicillin–streptomycin solution (Gibco BRL, Cat# 15140-122), 50 µg/mL ascorbic acid (Sigma-Aldrich, Cat# A7506), 1.5 g/L sodium bicarbonate (Sigma-Aldrich, Cat# S5761), and 1% nonessential amino acid solution (Gibco BRL, Cat# 11140-035); the cells were incubated at 37 °C in a humidified environment with 5% CO_2_. The charcoal-stripped fetal bovine serum was prepared by incubating 0.25% activated charcoal and 0.0025% dextran T-70 overnight at 4 °C and then by centrifuging the mixture at 500× *g* for 10 min at 4 °C. The supernatant was filtered and added to the bone medium. The D1 cells were subcultured using TrypLETM Express (Gibco BRL, Cat# 12604-013) until they reached 70–80% confluence. When they reached 70–80% confluence, the cells were treated with 50 μM IS (Sigma-Aldrich, Cat# A7506). In some experiments, the cells were pretreated (4 h prior to the experiments) with the AhR antagonist CH223191 (10 μM; Sigma-Aldrich, Cat# C8124).

### 4.2. Alizarin Red S Staining

The D1 cells were cultured in a bone medium for 3 days and then transferred to osteoinduction medium (bone medium + 5 mM β-glycerophosphate disodium salt hydrate [Sigma-Aldrich, Cat# G9422] and 50 nM dexamethasone [Sigma-Aldrich, Cat# D2915]). The medium was replenished every 2 to 3 days. Subsequently, the cells were stained with ARS. Specifically, the cells were fixed in 4% formalin–saline for 10 min, washed, and stained with 400 μL of 2% ARS solution in a 24-well plate. The stain was then removed through washing and the plates were air-dried. Mineralization was assessed by dissolving ARS in 10% acetic acid and performing spectrophotometric measurements at 450 nm.

### 4.3. Quantitative Real-Time Polymerase Chain Reaction

Total RNA was extracted from the D1 cells by using TRIzol (Sigma-Aldrich, Cat# 93289) and reverse-transcribed (2 μg) into complementary DNA by using the RevertAid RT Reverse Transcription Kit (Thermo Fisher Scientific, Cat# K1691). The expression levels of mouse *Runx2*, *Bmp2*, *Alp*, *Oc*, *Cyp1A1*, *Cyp1B1*, *Notch1–4*, *Hes1*, *Hey1*, and *GAPDH* were measured using specific primer pairs (Table S1) and quantified using the ChamQ Universal SYBR qPCR Master Mix (Vazyme Biotech Co., Ltd., Nanjing, China, Cat# Q711) and a PCR system (Applied Biosystems, Waltham, MA, USA). *GAPDH* was used as the reference gene. Fold changes in mRNA expression were calculated using the 2^−ΔCt^ method and compared against those in control cells. The PCR protocol was as follows: initial denaturation at 98 °C; 40 cycles of denaturation at 98 °C, annealing at 60 °C, and extension at 72 °C; and final extension at 72 °C. All PCR tests were run in triplicate.

### 4.4. Western Blotting

The D1 cells were lysed through incubation in an ice-cold radioimmunoprecipitation assay buffer at 4 °C for 10 min. After centrifugation at 14,000× *g* for 15 min, protein samples were prepared using a 5× loading buffer. The samples were boiled for 8–10 min and subsequently separated on a 10% sodium dodecyl sulfate polyacrylamide gel. Protein bands were transferred onto a nitrocellulose membrane. The membrane was blocked with 5% skim milk–Tris-buffered saline with 0.1% Tween 20 for 1 h. After washing, the membrane was incubated overnight at 4 °C with primary antibodies against Runx2 (Abcam, Cambridge, UK, Cat# ab23981), Bmp2 (Abcam, Cat# ab14933), Alp (Abcam, Cat# ab203106), Oc (Abcam, Cat# ab93876), AhR (Santa Cruz Biotechnology, Inc., Dallas, TX, USA, Cat# sc-133088), Hes1 (NOVUS Biologicals, Centennial, CO, USA, Cat# NBP1-47791), and cleaved-Notch1 (Elabscience Biotechnology Co., Ltd., Wuhan, China, Cat# E-AB-30054). Protein bands were detected using corresponding horseradish peroxidase-conjugated secondary antibodies and visualized using an enhanced chemiluminescence agent (Pierce; Rockford, IL, USA). Images were analyzed using ImageJ.

### 4.5. Measurement of Cytoplasmic and Nuclear AhR Concentrations

Separated cytoplasmic and nuclear AhR samples were analyzed through sodium dodecyl sulfate-polyacrylamide gel electrophoresis and Western blotting using antibodies. The D1 cells were harvested 2 and 4 h after IS treatment and then washed thrice with cold phosphate-buffered saline. Next, the cells were processed using a nuclear–cytosol extraction kit (Thermo Fisher Scientific, Cat# 78833) in accordance with the manufacturer’s instructions. The raw level of AhR was normalized to that of histone-H3 (Elabscience, Cat# E-AB-22081) to measure the nuclear concentration of AhR.

### 4.6. Immunofluorescence Staining

The D1 cells were washed with phosphate-buffered saline, fixed with 4% paraformaldehyde, and permeabilized with 0.1% Triton X-100. Subsequently, the cells were blocked with 1% bovine serum albumin and incubated overnight at 4 °C with primary antibodies against the AhR. The cells were then washed and incubated with goat antimouse immunoglobulin (Ig)G antibodies (Proteintech Group, Inc., Rosemont, IL, USA, Cat# sa00003-1). The nuclei were next stained using 4′,6-diamidino-2-phenylindole (Thermo Fisher Scientific). After washing, the cells were mounted and observed under a confocal fluorescence microscope (Zeiss, Oberkochen, Germany). A total of 10 image frames were randomly captured per culture well.

### 4.7. Ahr Knockdown

The D1 cells were transfected with siRNA against AhR (sequence: 5′-GGACGAGAGCAUUUACAGAAGCGAA-3′; Topgen, Taiwan) at the final concentration of 20 nM by using the TransIT-X2 Dynamic Delivery System (Mirus Bio LLC, Madison, WI, USA, Cat# MIR6000) in accordance with the manufacturer’s protocol. Experiments were performed 48 h after transfection. The level of *Ahr* knockdown was measured through Western blotting.

### 4.8. Luciferase Assay

Bmp2-Luc and Runx2-Luc plasmids were constructed using PCR fragments of various lengths—Bmp2-prom-2304bp, Bmp2-prom-230bp, and Bmp2-prom-223bp for Bmp2 and Runx2-prom-1297bp, Runx2-prom-217bp, and Runx2-prom-210bp for Runx2. The analysis focused on the presence of Hes1-binding elements. These fragments were cloned into the pGL4.17 vector (GeneCopoeia, Rockville, MD, USA), which carries the firefly luciferase gene (Topgen). The D1 cells were cotransfected with Bmp2 or Runx2 luciferase reporter plasmids (2 μg per 35-mm dish), a negative control, and the TransIT-X2 Dynamic Delivery System (Mirus, Cat# MIR6000) for 24 h. After 48 h, luciferase activity was measured through a dual-luciferase reporter assay (Vazyme) in accordance with the manufacturer’s instructions.

### 4.9. Chromatin Immunoprecipitation

The D1 cells were treated with IS for 1 day or left untreated. Subsequently, the cells were treated with 1% formaldehyde for 15 min. Cross-linked chromatin was then prepared and sonicated to, on average, 500-bp fragments. The fragments were subjected to a ChIP assay (Abcam, Cat# ab500) performed in accordance with the manufacturer’s guidelines. In brief, the samples were incubated overnight at 4 °C with anti-Hes1 antibodies (NOVUS) or negative control antibodies (rabbit IgG [Invitrogen, Carlsbad, CA, USA, Cat# 25-4714-80]). Equal amounts of immunoprecipitated DNA and relevant controls were subjected to quantitative PCR. The results were normalized to the total chromatin input as follows: 2^−ΔCt^ × 100 (% DNA input). The sequences of primers used in the ChIP assay are presented in Supplementary Table S1.

### 4.10. Statistical Analysis

Data are presented as the mean ± standard deviation derived from at least three consistent experiments. Comparisons between two groups were performed using an unpaired *t*-test. By contrast, comparisons among three or more groups were performed using a one-way analysis of variance, followed by the Tukey post hoc test. Real-time PCR outliers were excluded on the basis of high z scores or inconsistent melting curve peaks. A *p*-value of <0.05 was deemed significant. GraphPad Prism 6.02 Software was used to perform statistical analysis.

## Data Availability

The original contributions presented in the study are included in the article/Supplementary Materials, further inquiries can be directed to the corresponding authors.

## Supplementary Materials

The following supporting information can be downloaded at https://www.mdpi.com/article/10.3390/ijms25168770/s1.
