# Supplementary material for: Indoxyl Sulfate Inhibits Osteogenesis in Bone Marrow Mesenchymal Stem Cells through the AhR/Hes1 Pathway
Source: Int J Mol Sci. 2024 Aug 12;25(16):8770. doi: 10.3390/ijms25168770 (PMC11354967; doi:10.3390/ijms25168770)
Supplement: Supplementary file 1 [file ijms-25-08770-s001.zip › Supplement figures.docx]

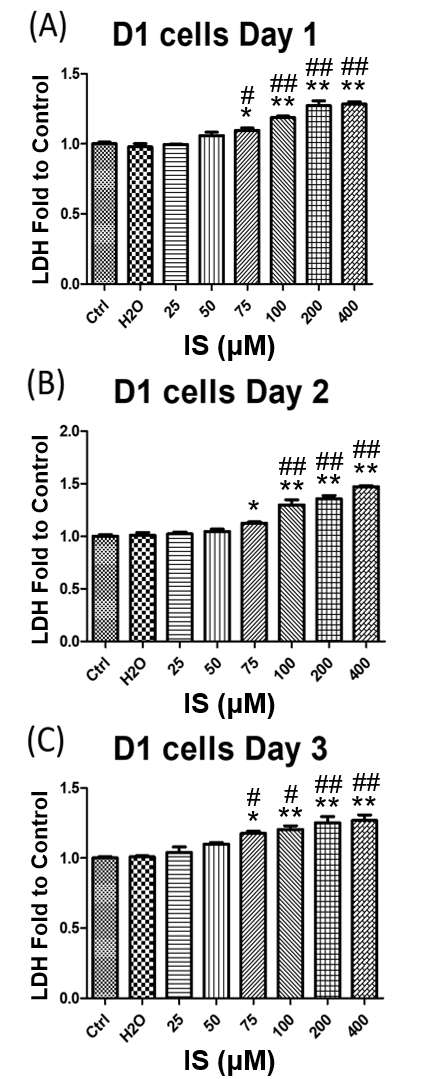


**Figure S1.** Cytotoxicity of indoxyl sulfate (IS) treatment on D1 cells evaluated by lactate dehydrogenase (LDH) cytotoxicity assay. LDH leakage was measured in each culture and expressed as fold change compared to the control. D1 cells were treated with various concentrations of IS, H_2_O, or left untreated (control) for different durations: 1 day (A), 2 days (B), and 3 days (C). The H_2_O control was included to provide a baseline reference and to ensure that any observed effects were due to the IS treatment rather than the solvent. Data are presented as mean ± standard deviation from three independent experiments (*p < 0.05, **p < 0.01; *compared with control, #compared with H_2_O; analysis of variance). Ctrl, control.


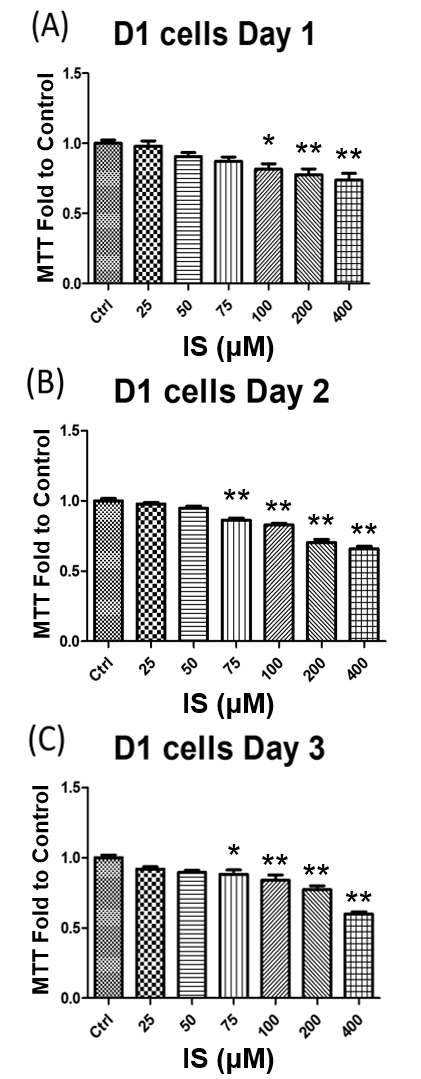


**Figure S2.** Effect of IS treatment on D1 cell viability as evaluated by 3-[4,5-dimethylthiazol-2-yl]-2,5-diphenyl-tetrazolium bromide (MTT) assay. D1 cells were treated with various concentrations of IS or left untreated (control) for different durations: 1 day (A), 2 days (B), and 3 days (C). Data are presented as mean ± standard deviation from three independent experiments (*p < 0.05, **p < 0.01; *compared with control; analysis of variance). Ctrl, control.


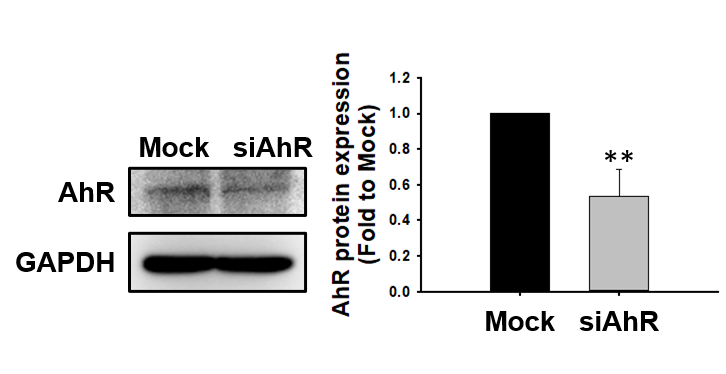


**Figure S3.** Efficiency of AhR knockdown in D1 cells transfected with vectors encoding control-siRNA or AhR-siRNA for 48 h. Data are presented as mean ± standard deviation values (*p < 0.05, **p < 0.01; *compared with mock; Student t test). AhR, aryl hydrocarbon receptor.


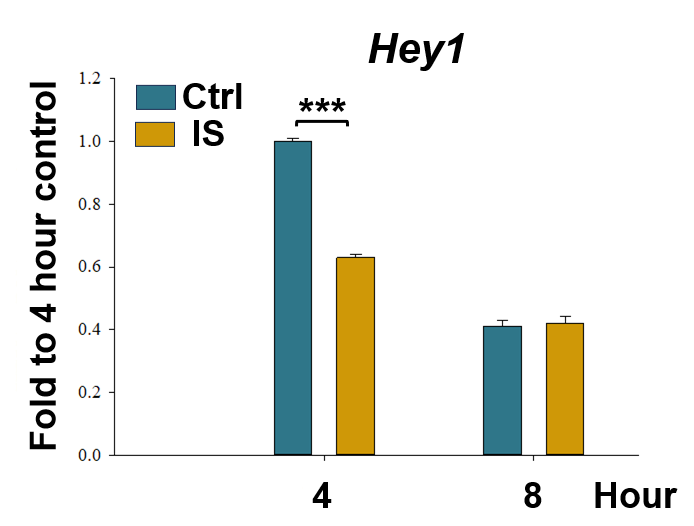


**Figure S4.** Quantitative real-time polymerase chain reaction (qRT-PCR) analysis of mRNA levels of hairy/enhancer of split with YRPW motif 1 (Hey1) in D1 cells. D1 cells were treated with IS for 4 hours and 8 hours or left untreated as controls. The results show that Hey1 mRNA levels did not change significantly in D1 cells treated with IS for 8 hours compared to the control. Data are presented as mean ± standard deviation values (*p < 0.05, **p < 0.01, and ***p < 0.001; *compared with control; Student t test). Ctrl, control.
