# Supplementary material for: Indoxyl Sulfate Inhibits Osteogenesis in Bone Marrow Mesenchymal Stem Cells through the AhR/Hes1 Pathway
Source: Int J Mol Sci. 2024 Aug 12;25(16):8770. doi: 10.3390/ijms25168770 (PMC11354967; doi:10.3390/ijms25168770)
Supplement: Supplementary file 1 [file ijms-25-08770-s001.zip › Supplementary figures.pptx]

## Slide 1
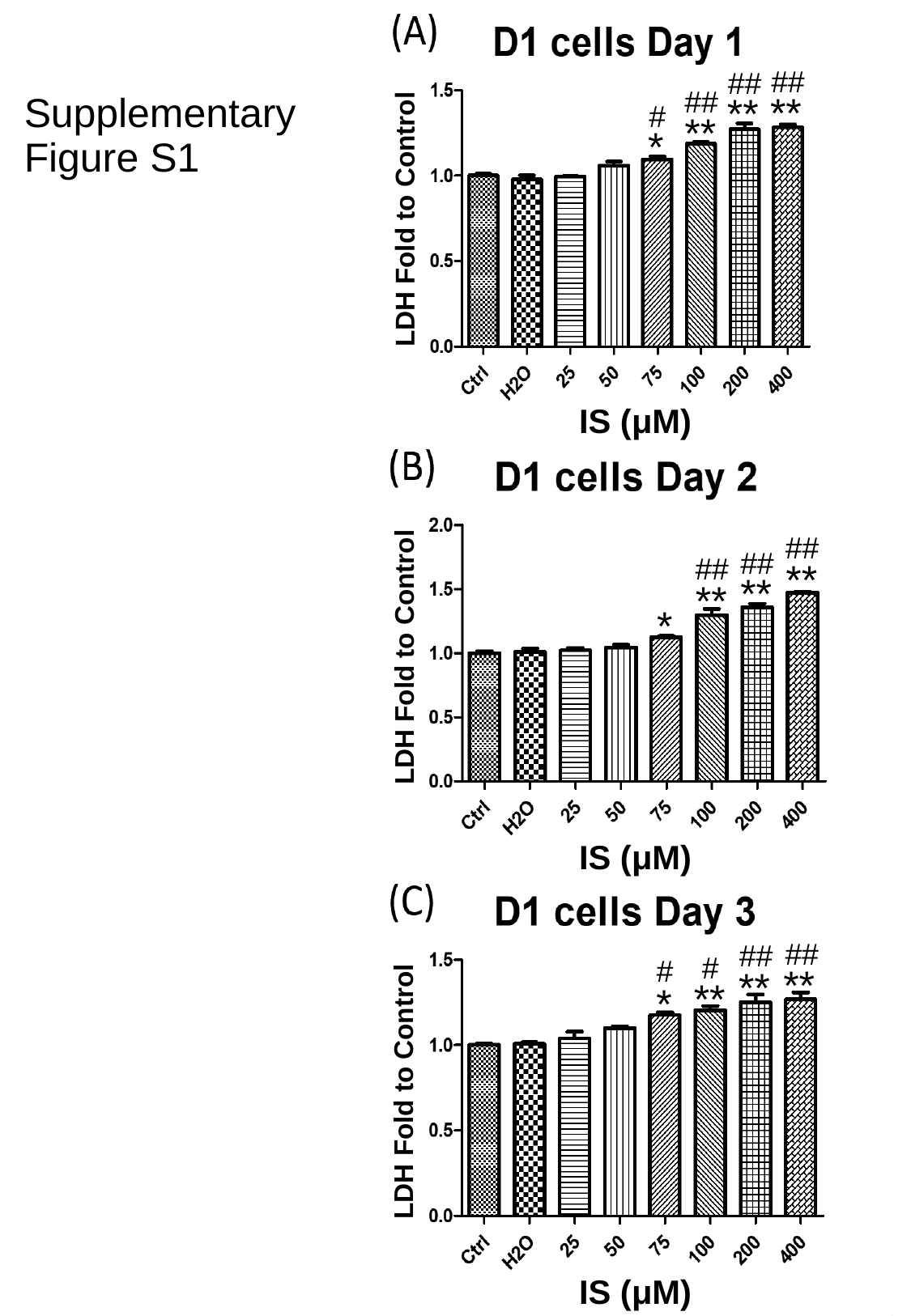

##
##
##
**
**
#
**
*
LDH Fold to Control
| Supplementary Figure S1 |
| --- |
IS (μM)
##
##
##
**
**
**
*
LDH Fold to Control
IS (μM)
##
##
#
#
**
**
**
*
LDH Fold to Control
IS (μM)
IS (μM)

## Slide 2
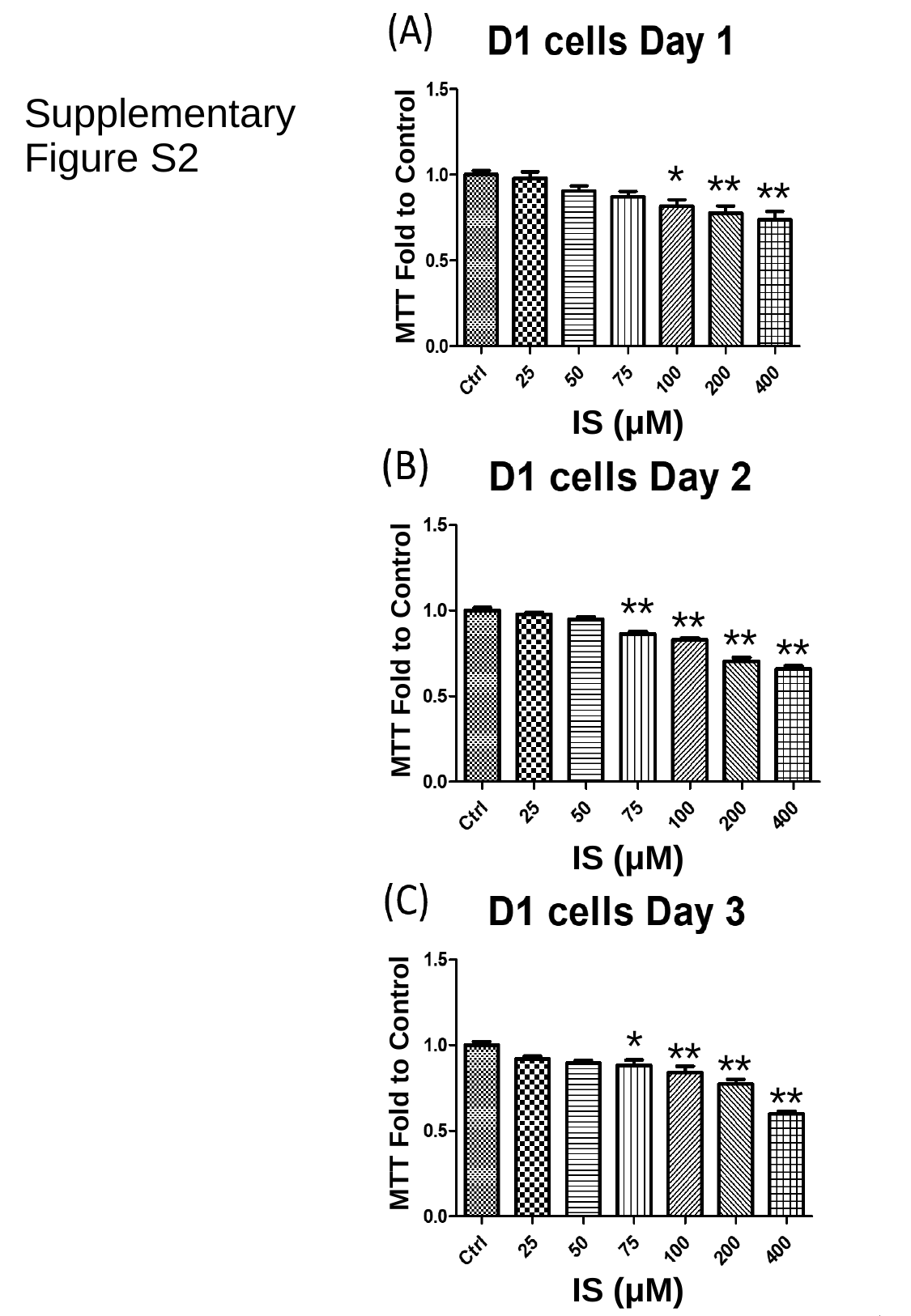

MTT Fold to Control
*
**
**
| Supplementary Figure S2 |
| --- |
IS (μM)
**
**
**
**
MTT Fold to Control
IS (μM)
*
**
**
MTT Fold to Control
**
IS (μM)

## Slide 3
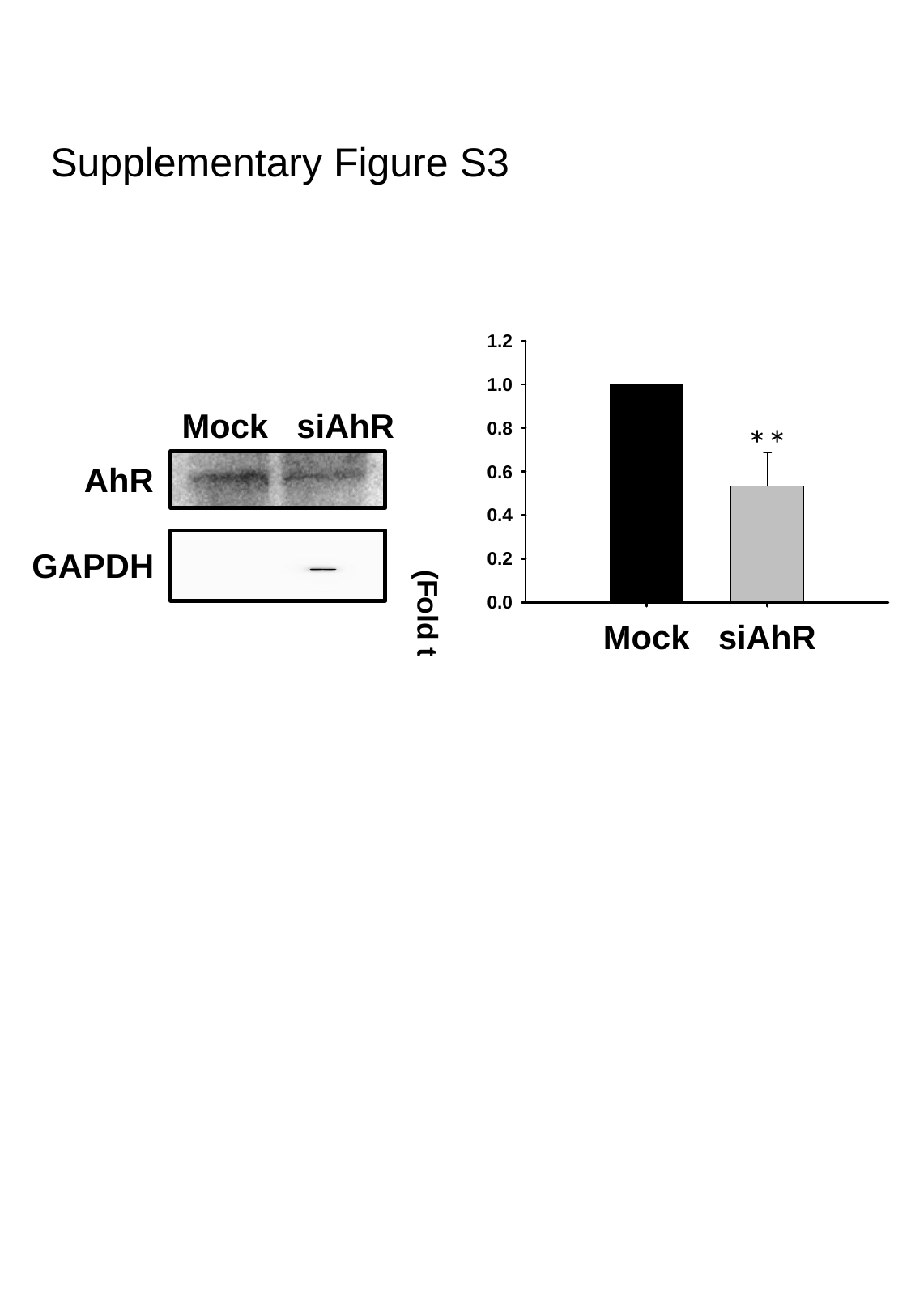

| Supplementary Figure S3 |
| --- |
siAhR
Mock
**
AhR
GAPDH
siAhR
Mock

## Slide 4
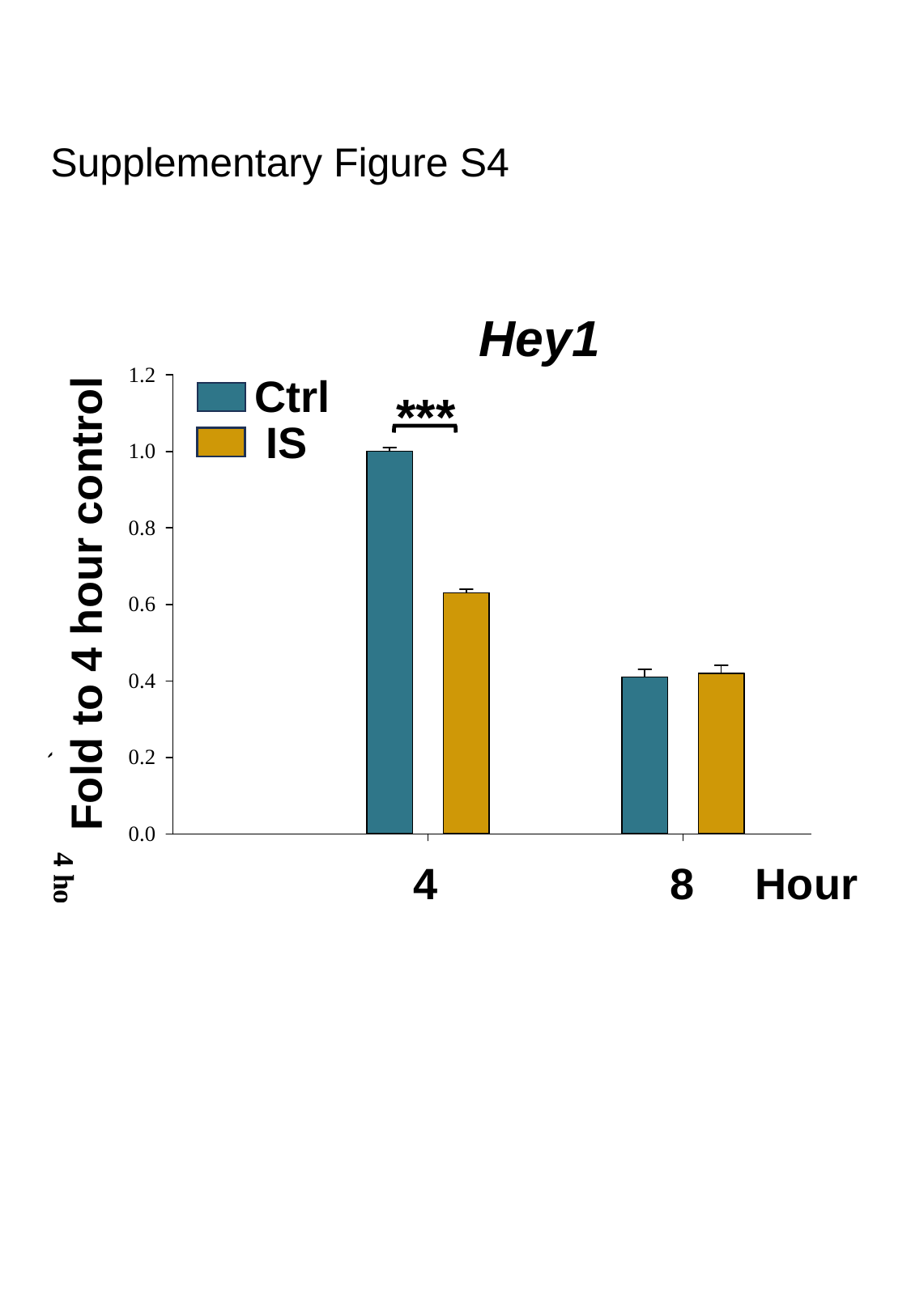

| Supplementary Figure S4 |
| --- |
Hey1
Ctrl
***
IS
Fold to 4 hour control
4
8
Hour
