# Supplementary material for: Indoxyl Sulfate Inhibits Osteogenesis in Bone Marrow Mesenchymal Stem Cells through the AhR/Hes1 Pathway
Source: Int J Mol Sci. 2024 Aug 12;25(16):8770. doi: 10.3390/ijms25168770 (PMC11354967; doi:10.3390/ijms25168770)
Supplement: Supplementary file 1 [file ijms-25-08770-s001.zip › Supplementary Table 1.docx]

Table S1: Primers used in this study. The primers were synthesized by Sangon Biotech and presented as follows:

| Gene | Direction | Sequence |
| --- | --- | --- |
| *Bmp2* | Forward  Reverse | 5'-TGTACCGCAGGCACTCAGG-3'  5'-CACTCATCTCTGGAAGTTCCTCC-3' |
| *Runx2* | Forward  Reverse | 5'-CACCTCTGACTTCTGCCTCTG-3'  5'-GGATGAAATGCTTGGGAACTGC-3' |
| *Alp* | Forward  Reverse | 5'-GGCTGGAGATGGACAAATTCC-3'  5'-CACACAAGTAGGCAGTGGCAGT-3' |
| *Oc* | Forward  Reverse | 5'-CAATAAGGTAGTGAACAGACTCCG-3'  5'-TGATAGCTCGTCACAAGCAGG-3' |
| *Cyp1A1* | Forward  Reverse | 5'-CTCTTCCCTGGATGCCTTCA -3'  5'-TCAATGAGGCTGTCTGTGATGTC-3' |
| *Cyp1B1* | Forward  Reverse | 5'-AGCCAGGACACCCTTTCCA-3'  5'-CCCACAACCTGGTCCAACTC-3' |
| *Notch1* | Forward  Reverse | 5'-TGCCTGTGCACACCATTCTGC-3'  5'-CAATCAGAGATGTTGGAATGC-3' |
| *Notch2* | Forward  Reverse | 5'-ATGCACCATGACATCGTTCG-3'  5'-GATAGAGTCACTGAGCTCTCG-3' |
| *Notch3* | Forward  Reverse | 5'-TTGGTCTGCTCAATCCTGTAGC-3'  5'-TGGCATTGGTAGCAGTTGCTG-3' |
| *Notch4* | Forward  Reverse | 5'-AAGCGACACGTACGAGTCTGG-3'  5'-ATAGTTGCCAGCTACTTGTGG-3' |
| *Hes1* | Forward  Reverse | 5'- CGGTCTACACCAGCAACAGT-3'  5'-AGGCGCAATCCAATATGAAC-3' |
| *Hey1* | Forward  Reverse | 5'-CGACGAGACCGAATCAATAAC-3'  5'-CAAACTCCGATAGTCCATAGCC-3' |
| *GAPDH* | Forward  Reverse | 5'-AATGTGTCCGTCGTGGATCTG-3'  5'-GCCCAAGATGCCCTTCAGT-3' |
